# Quasi-periodic ripples in high latitude electron content, the geomagnetic field, and the solar wind

**DOI:** 10.1038/s41598-019-57201-4

**Published:** 2020-01-28

**Authors:** M. J. Birch, J. K. Hargreaves

**Affiliations:** 1 Jeremiah Horrocks Institute for Mathematics, Physics and Astronomy, University of Central Lancashire, Preston, UK; 2 Department of Physics, Lancaster University, Lancaster, UK

**Keywords:** Magnetospheric physics, Solar physics

## Abstract

In this study we present evidence for the existence of quasi-periodic ripples in the F-region electron content at high latitude, the magnetic flux density at geosynchronous orbit, and the solar wind dynamic pressure and IMF flux density at L1. The ripples are shown to be continually present during all 14 days of observations in different years and seasons, and at all local times, though they are generally small in magnitude relative to the background. The frequencies of all the parameters are, on average, remarkably similar, and the amplitudes of the selected peaks of some parameters show strong correlations for which regression formulae are given. These results support the proposition that the ripples propagate from the solar wind to the F-region, and that they are a related, persistent phenomenon.

## Introduction

Spatial and temporal structuring in the F-region of the ionosphere is a well-known phenomenon which has been observed on many occasions. The structures usually extend along the geomagnetic field and are, therefore, almost vertical at high latitudes.

This paper is based on observations of quasi-periodic electron density structures in the high latitude F-region using the EISCAT Svalbard radar. A previous paper^1^, covering 24 hours of observations over 4 days in February and March 2015, showed the presence of F-region electron density structures with a median periodicity of about 22 minutes. The present investigation extends the scope of this study to include a further 10 days of electron density data in different years and seasons. Similar structures are reported in the geomagnetic field, and in the solar wind particle flux and IMF flux density. The relationship between these structures is investigated, and evidence for their origin is discussed.

## Electron Content Structures

### EISCAT Svalbard observations

In late February and early March of 2015, four special programme runs were made with the 42 m EISCAT Svalbard radar (ESR) at Longyearbyen (78.153°N, 16.029°E, L-value 15.8). The runs were from 17:00 to 23:00 UT on February 27 and 28, and from 05:00 to 11:00 UT on March 1 and 2, each covering 6 hours up to approximately midnight or noon in local magnetic time at the observing site. The ESR observations of electron density covered heights from about 30 to 504 km at 1 minute time resolution. The antenna was aligned along the geomagnetic field, at 8.4° from the vertical and at azimuth 184.5°, the half-power beamwidth being ±0.3°.

**Figure 1:**
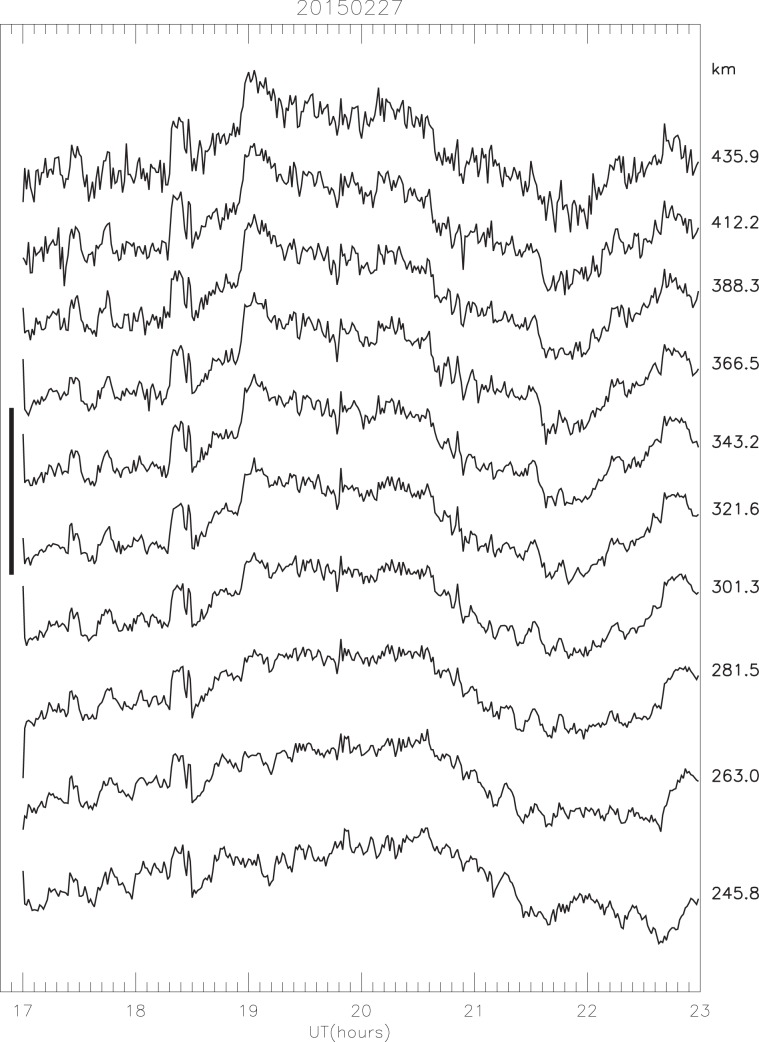
Electron density variations on a log scale (1-minute data) in the F-region on February 27 at the indicated heights. The vertical bar shows a factor of 10 variation in electron density. (Reproduced from Fig. 9 in Hargreaves and Birch^1^).

Between about 250 and 450 km (F-region) the variability in electron density is remarkably similar at all heights, though the day-to-day variation at a given height may be as much as a factor of 20. Taking the observations of February 27 as an example, the correlation coefficient between adjacent pairs of heights about 20 km apart is only once less than 0.9, and for a vertical separation of 154 km it is still 0.86 (Fig. 1). The electron content along the radar beam over the height range 237.50 to 354.85 km (the range within which the structures are most similar and free from noise) is derived by integrating the electron density in “slabs” centred on heights at 228, 245, 262, 281, 300, 320, 342 and 365 km (mean slab thickness about 20 km).

**Figure 2:**
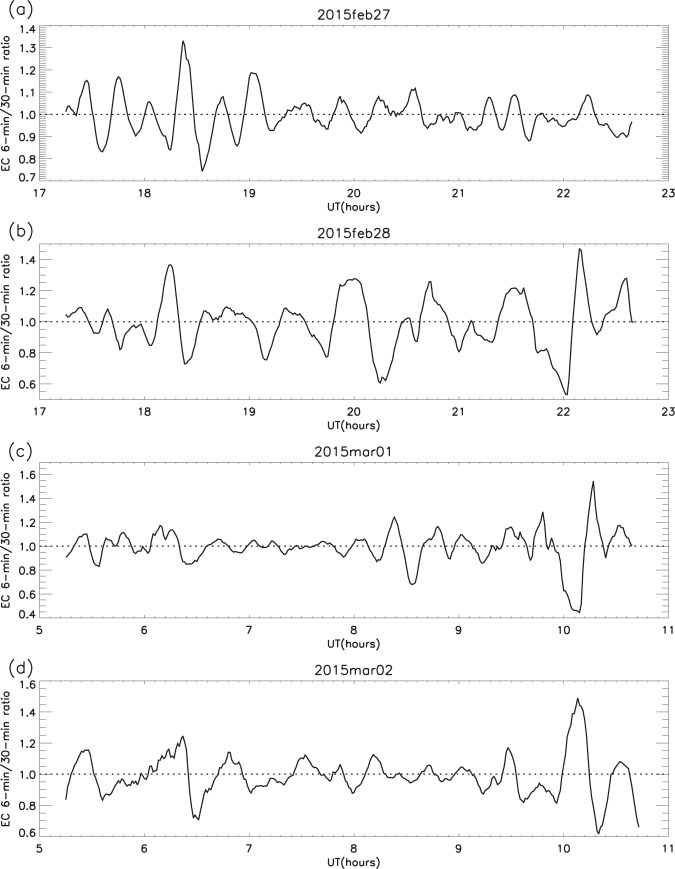
Electron content (EC) ratio from 237.5 to 354.85 km: 2015 (**a**) February 27; (**b**) February 28; (**c**) March 1; (**d**) March 2. (Determined by taking the ratio of 6-min to 30-min smoothing, to remove both short-term noise and long-term variations. Reproduced from Fig. 10 in Hargreaves and Birch^1^).

**Figure 3:**
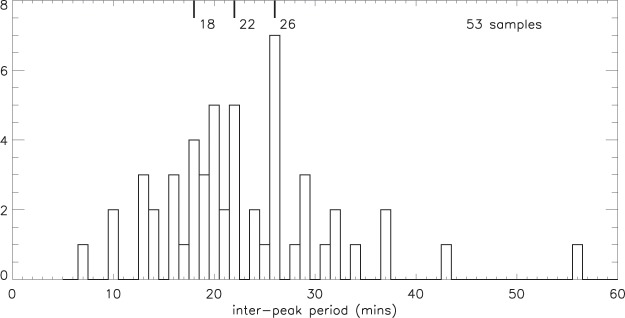
Inter-peak periods of 42 m electron content ratio from Fig. 2, for all 4 days: Feb 27–28, Mar 1–2 2015 (showing median and quartiles). (Reproduced from Fig. 11e in Hargreaves and Birch^1^).

To reduce the noise component, and to remove long-term trends, a smoothing procedure was used in which the 1-minute data were averaged over 6 minutes and this value was then divided by the average over 30 minutes, giving the electron content variations as a “ratio”. (Refer to *Validation of the smoothing procedure*, comments regarding the efficacy of spectral analysis as an alternative approach, and the testing of this method using random data.) Using this smoothing procedure, Fig. 2 shows the variations in the electron content ratio derived for all four days over the height range 237.50 to 354.85 km; the variations appear to be quasi-periodic in nature. Peaks with magnitudes ≥1.03 were selected (see *Selection criteria*), and the 53 intervals between the 57 selected peaks are summarised in a histogram (Fig. 3). (Throughout this analysis, for any given set of data, there are occasional data gaps or intervals of bad data. Consequently, if *n* is the number of peaks sampled, the number of inter-peak periods can be less than *n* − 1). The median inter-peak period over all four days is 22 minutes, with quartiles at 18 and 26 minutes (Table 1a), and the daily medians are in the range 20–26 minutes. It is not obvious whether there was a dynamic component to the structures because these observations were made with a single, field-aligned radar beam.

**Table 1:** Summary of the periodicity statistics for (a,b) electron content, (c) geomagnetic flux density, (d) solar wind dynamic pressure and total IMF flux density (CP: common programme; LQ: lower quartile; UQ: upper quartile; IQ: inter-quartile).

Date	No of samples (hrs)	Median (mins)	LQ (mins)	UQ (mins)	IQ range (mins)
**(a) 42 m F-region electron content**					
2012 Dec 11–14	152 (77)	**25**	19	34	15
2015 Feb 27–28,Mar 1–2	53 (24)	**22**	18	26	8
2015 Mar 23–24	44 (28)	**28**	20	34	14
2017 Sep 6–9	77 (53)	**29**	21	40	19
All 14 days	326 (182)	**25**	19	34	15
All 10 days (CP data)	276 (158)	**26**	20	38	18
**(b) 32 m F-region electron content**					
2012 Dec 11–14	150 (77)	**28**	22	37	15
2015 Mar 23–24	42 (28)	**26**	22	33	11
2017 Sep 6–9	85 (53)	**28**	19	43	14
All 10 days (CP data)	273 (158)	**27**	21	38	17
**(c) Geomagnetic flux density (GOES13,15)**					
2012 Dec 11–14	187, 186 (96)	**27, 27**	20, 20	35, 36	15, 16
2015 Feb 27–Mar 24	303, 301 (144)	**25, 25**	21, 20	35, 33	14, 13
2017 Sep 6–9	183, 164 (96)	**28, 31**	23, 24	35, 38	12, 14
All 14 days (00–24 LT)	673, 651 (336)	**26, 27**	20, 20	34, 35	14, 15
All 14 days (10–14 LT)	111, 98	**25, 23**	19, 20	31, 35	12,15
All 14 days (22–02 LT)	95, 88	**28, 28**	22, 23	36, 38	15,15
**(d) Solar wind (P**_***dyn***_**, B**_***tot***_**)**					
2012 Dec 11–14	89 (44), 176 (86)	**23, 26**	18, 20	31, 34	13, 14
2015 Feb 27–Mar 24	106 (66), 196 (112)	**26, 24**	20, 20	31, 33	11, 13
2017 Sep 6–8	59 (31), 156 (68)	**23, 23**	17, 19	31, 29	14, 10
All 13 days	253 (141), 528 (266)	**25, 24**	19, 20	31, 32	12, 12

### Extension using ESR common programme data

#### 42 m antenna

To extend the study, more Svalbard observations were obtained from the EISCAT common programme library, and a further 10 days of field-aligned 42 m data were selected for analysis: December 11–14 2012, March 23–24 2015, and September 6–9 2017 (the antenna alignment for these days being the same as in *EISCAT Svalbard observations*). These periods are near to the winter solstice and to the spring and autumn equinoxes. Together with the 4 days of observations in *EISCAT Svalbard observations*, this gives 14 days (182 hours) in total. Geomagnetic activity ranged from quiet (K_*p*_ = 0) to severe (K_*p*_ = 8+) during these days. The same smoothing procedure was used to determine the electron content ratios for each of these 10 days. It was found that the fluctuations (or “ripples”) occur at all times of day, though varying in magnitude. The medians and quartiles of their inter-peak periods are shown in Table 1a. The new results are not inconsistent with those of the first set of observations (*EISCAT Svalbard observations*), suggesting that the quasi-periodic ripples are a general feature of the F-region at this latitude (L-value 15.8).

#### 32 m antenna

**Figure 4:**
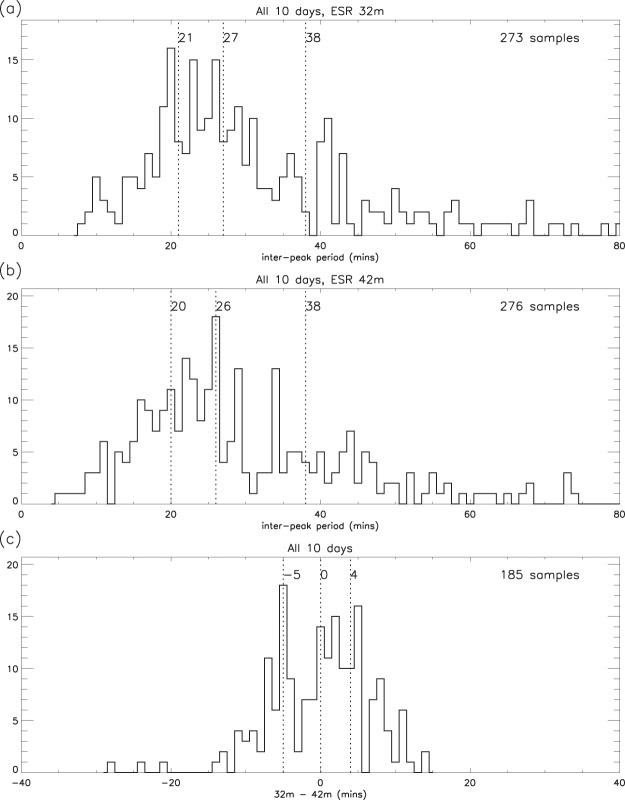
Comparison of the statistics (median, quartiles and sample sizes) for the ESR common programme data: (**a**) 32 m inter-peak periods; (**b**) 42 m inter-peak periods; (**c**) time difference between selected pairs of peaks.

The common programme data also include simultaneous observations with the 32 m antenna fixed at 30° elevation and −29.1° azimuth, the periods of observation being the same as those for the 42 m antenna. The average inter-peak period of the electron content ratios for the 32 m antenna agrees very closely with that for the 42 m (Fig. 4(a,b), Table 1b). The median difference between the times of selected pairs of 32 m and 42 m peaks is negligible, 50% of the samples being within about 5 minutes (Fig. 4c) of each other, and tests show that there is no local time dependence, the noon and night sectors giving very similar distributions.

**Figure 5:**
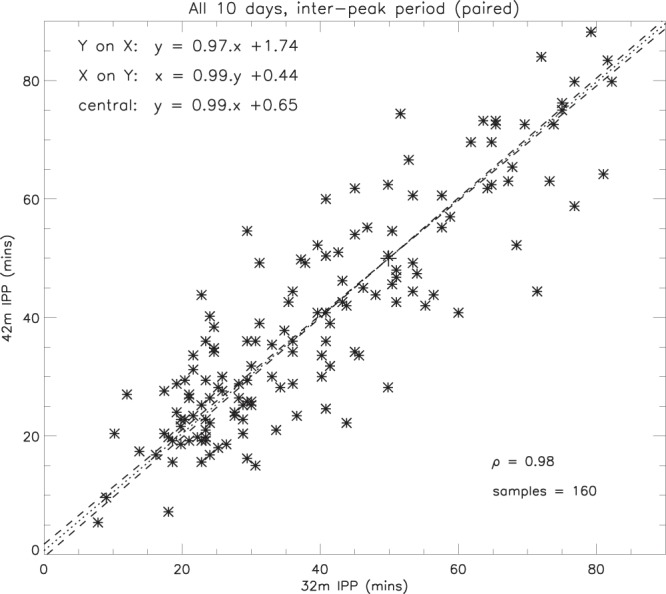
Comparison of selected pairs of inter-peak periods for the 10 days of 32 m and 42 m common programme data, showing regression lines, correlation coefficient (*ρ*), and sample size (IPP = inter-peak period).

Furthermore, a comparison of selected pairs of near-coincident inter-peak periods (Fig. 5) shows very good agreement between the two antennae, the correlation coefficient (*ρ*) between the periodicities being 0.98 for 160 samples. (Of the 253 32 m and 276 42 m inter-peak periods in Fig. 4(a,b), only 160 pairs could be correlated in Fig. 5 without ambiguity). The regression equation of the central line is given by -

1 $$R{p}_{42}=0.99R{p}_{32}+0.65$$

where *Rp*_42_ is the inter-peak period of the 42 m electron content ripples, and *Rp*_32_ is the inter-peak period of the 32 m ripples (Table 2). This indicates that, on average, the variations in periodicity measured by both antennae are equal within 1%, with a constant offset of less than 1 minute.

**Table 2:** Summary of the central line equations, correlation coefficients, sample sizes, and Fisher z-transform statistics for the regression relations in this study.

Figure	Central line	*ρ*	No of samples	z	SE(z)	z/SE(z)
5	Rp_42_ = 0.99 *Rp*_32_ + 0.65	0.98	160	2.298	0.0798	29.0
15	${{\rm{R}}}_{42}^{\ast }$ = 1.24 (${{\rm{R}}}_{32}^{\ast }$)^1.07^	0.48	163	0.523	0.0791	6.7
16	${{\rm{G}}}_{15}^{\ast }$ = 1.38 (${{\rm{G}}}_{13}^{\ast }$)^1.03^	0.78	457	1.045	0.0469	22.3
17	G* = 0.0008(A_*p*_)^1.06^	0.75	790	0.973	0.0356	27.3
18	G* = 0.51(P*)^1.62^	0.71	251	0.887	0.0635	14.0
19	G* = 0.26(B*)^1.28^	0.26	268	0.226	0.0614	4.3

(Symbols: Rp = EC inter-peak period; R = EC peak magnitude; G = GOES peak magnitude; A_*p*_ = geomagnetic index; P = P_*dyn*_ peak magnitude; B = B_*tot*_ peak magnitude. According to the Fisher z-transform^27,28^, the probability of any of these correlations occurring by chance is less than 0.1% in every case. The values in column 7, z/SE(z), are converted to the probability estimate using a standard normal distribution table).

As explained above, the electron content along the radar beam is derived by integrating the electron density in “slabs” centred on heights at 228, 245, 262, 281, 300, 320, 342 and 365 km. In the case of the 32 m antenna, elevated at 30°, these heights correspond to distances from Svalbard in the range 395–674 km at the approximate azimuth −30°, and the peaks in 32 m electron content ratio are therefore derived from observations distributed over this range. This comparison shows that the ripples in electron content are not limited to Svalbard, but also extend a considerable distance towards the north-west.

#### Summary

From the results of the analysis of the ESR electron content it is clear that ripples, of median inter-peak period about 25 minutes (frequency 0.67 mHz) and inter-quartile range 15 minutes (1.11 mHz), are generally present in the F-region electron content at high latitude. These ripples are persistent in the vicinity of Svalbard and within about 674 km towards the north-west. Two questions now arise: how widespread are they and what is their origin?

## Geomagnetic Field Observations

The GOES spacecraft, in geosynchronous orbits at 6.6 Re, carry magnetometers which continually measure the geomagnetic flux density. GOES13 (at 75°W) and GOES15 (at 135°W) were in use during the years in question, and were separated by 4 hours in local time, the mean longitude being 105°W. (For comparison, the ESR is at about 15°E). The GOES magnetometer data have no significant gaps, so the total coverage for the 14 selected days is 336 hours (the total for the ESR over the same 14 days being 182 hours). Subsequently, the 2015 data (Feb 27–28, Mar 1–2, Mar 23–24) will be merged together, thus representing (approximately) the “spring equinox”.

**Figure 6:**
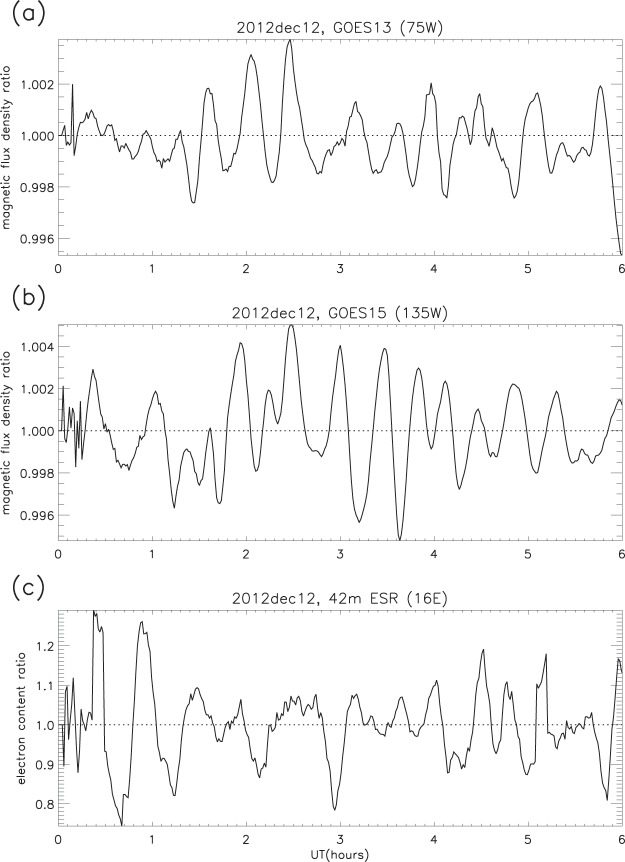
Geomagnetic flux density ratio: (**a**) GOES13, and (**b**) GOES15. (**c**) 42 m ESR electron content ratio. Dec 12 2012, 0000–0600 UT.

**Figure 7:**
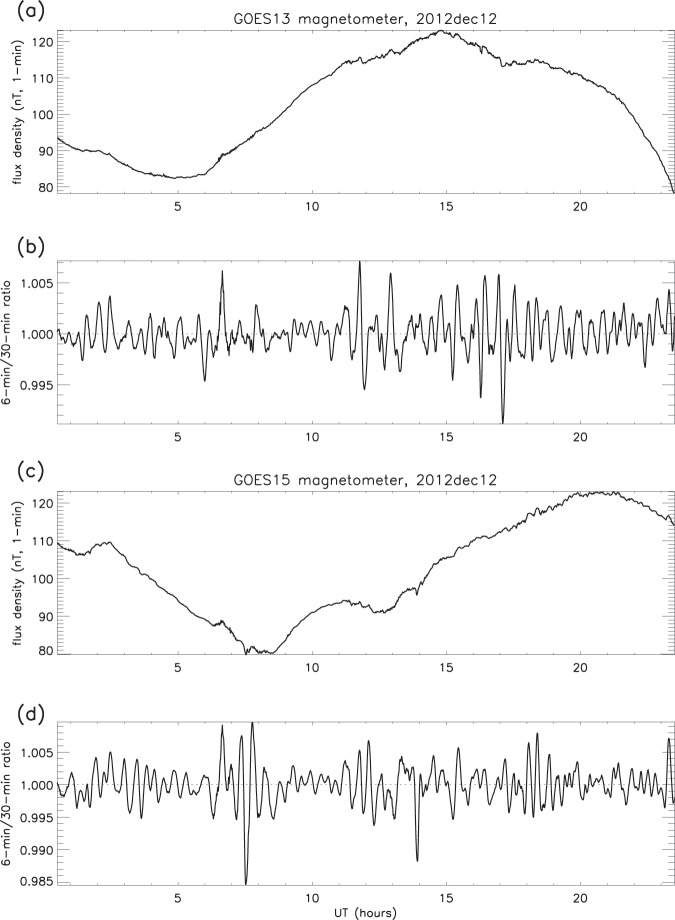
GOES magnetometer data on December 12 2012: (**a**) GOES13 magnetic flux density (original time series); (**b**) GOES13 ratios; (**c**) GOES15 magnetic flux density (original time series); (**d**) GOES15 ratios (examples).

The same smoothing procedure as that applied to the electron content was used to determine the geomagnetic flux density ratios, and plots show that similar behaviour occurs in the magnetosphere (for example, Fig. 6). (For each period of 1-minute data smoothed to 30 minutes (the denominator in the ratio procedure), the smoothing function leaves unaffected the first and last 15 minutes of each period. Subsequent plots omit these non-smoothed intervals.) It was found that the ripples in flux density ratio are both persistent and spatially widespread over all 14 days, and overall are smoother and more regular in structure than those in the electron content, though their relative amplitude is in general much smaller (as illustrated in Fig. 7, in which a direct comparison is given between the original 1-minute time series and the derived GOES ratios). Though similar structures are clearly present in both the F-region electron content and the geomagnetic field, they have a tendency to drift in and out of phase.

**Figure 8:**
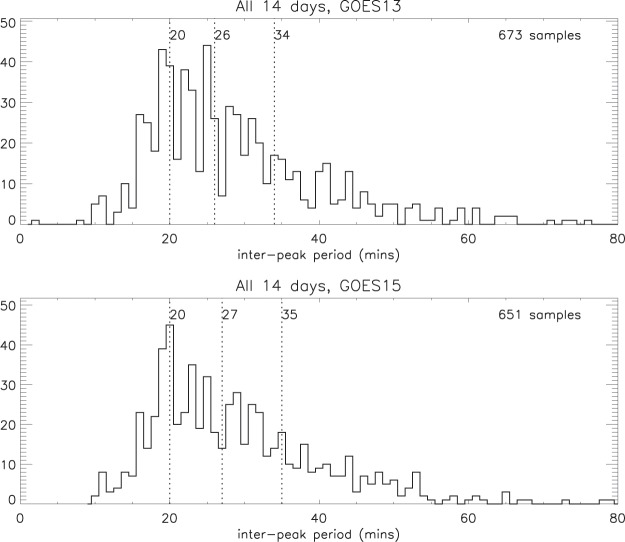
Statistics for GOES13 and GOES15 geomagnetic flux density ratio inter-peak periods for the 14 selected days (medians, quartiles and number of samples).

Because the amplitudes of the ripples (relative to the background) are generally much smaller in the geomagnetic flux density than in the electron content, clearly-defined peaks in the GOES 13 and 15 ratios were selected without applying a magnitude limit (see *Selection criteria*). The statistics of the inter-peak periods between these selections are summarised in Fig. 8 (for the 14 days combined) and in Table 1c (which also shows the separate “seasons”). There is clearly a distinct similarity between these results and those for the electron content ratio in Table 1(a,b), and, furthermore, the statistics are very similar for the two satellites.

### Time differences between associated peaks in electron content and geomagnetic flux density

**Figure 9:**
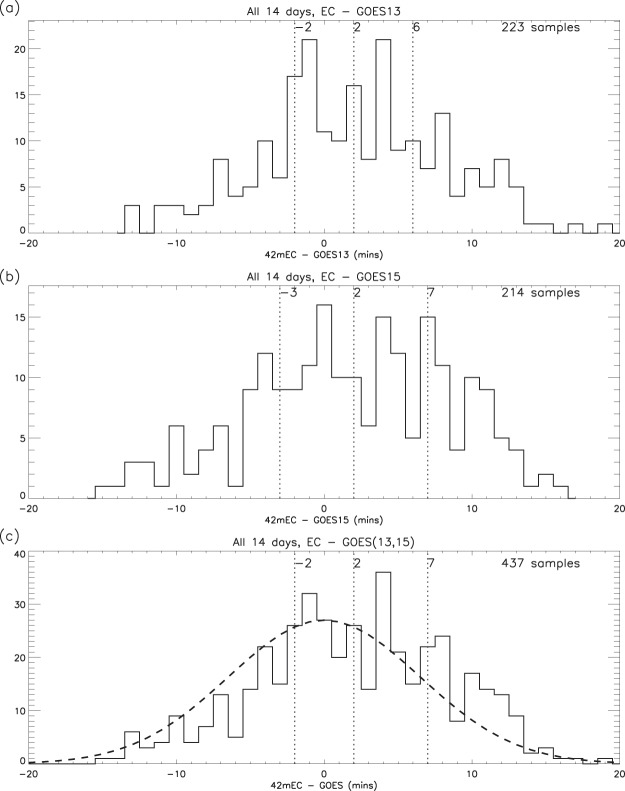
Time differences between selected peaks in geomagnetic flux density ratio and electron content ratio: (**a**) EC - GOES13; (**b**) EC - GOES15; (**c**) Combination of (**a**,**b**). The dashed line in (**c**) is the theoretical normal distribution of the data values based on the observed standard deviation (6.5) and an assumed zero mean time difference. (A positive delay indicates that the GOES peak precedes the associated peak in electron content).

By selecting associated peaks in the geomagnetic field and the 42 m electron content, their time difference can be determined, thus testing the principle of “cause and effect”. For instance, if the field fluctuations are driving those in the F-region electron content they would be expected to come first. However, the geosynchronous GOES13 and GOES15 spacecraft are separated in local time from the ESR antennae by 6 hours and 10 hours (respectively), which means that, on occasion, it is likely that peaks in the electron content will precede those in the geomagnetic field, particularly when the ESR is on the dayside. The statistics for the 14 days of observations are shown in Fig. 9, and it can be seen that this is indeed the case, the peaks in electron content being delayed by 2 minutes (on average) with respect to both GOES13 and 15 (as verified by the normal distribution test in Fig. 9c).

### Local time dependence of geomagnetic field ripples

**Test 1**. By dividing the inter-peak periods for each spacecraft into separate sectors for dayside (10–14 LT) and nightside (22-02 LT), the local time dependence of the geomagnetic field periodicity can be investigated. Table 1c shows the statistics for these sectors for all 14 days combined, and, on average, there is evidence of a reduction in the median and quartiles of 3 to 4 minutes on the dayside compared to the nightside.

**Figure 10:**
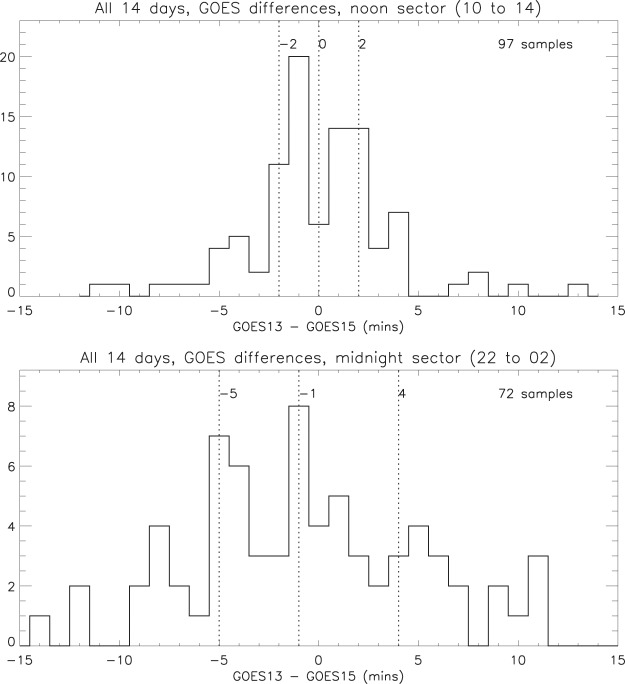
Time differences between selected pairs of GOES 13 and 15 peaks, for ±2 hours from local noon and midnight (medians, quartiles and number of samples).

**Test 2**. Using these same local time sectors (in this test, based on the mean LT of the two spacecraft), the time differences between selected pairs of GOES 13 and 15 peaks are summarised in Fig. 10. It is evident that the variability is greater on the nightside than on the dayside, the inter-quartile ranges being 9 minutes and 4 minutes respectively.

Together, these two LT-dependent tests indicate that the ripples in the geomagnetic field appear to be more coherent on the dayside, suggesting that the driving mechanism comes from the direction of the bowshock, not from the magnetotail. This is reasonable, because the ripples appear to be a continuous, persistent phenomenon and are most likely to be driven by the solar wind. Substorms may be considered as a possible driving force, but in fact they were infrequent during all but one of the selected days.

## Solar Wind Observations

Solar wind conditions are measured by the Wind and ACE spacecraft at the L1 Lagrangian point, about 1.5 × 10^6^ km sunwards from Earth (i.e. 1% of the Earth-Sun distance). Solar wind observations (dynamic pressure (P_*dyn*_), particle density, flow speed, and scalar magnetic field (B_*tot*_)) were provided by NASA GSFC (http://cdaweb.gsfc.nasa.gov/), pre-corrected in UT from L1 to Earth’s bowshock (for details of the algorithm see https://omniweb.gsfc.nasa.gov/html/HROdocum.html and references therein). The particle data have many bad data gaps, the P_*dyn*_ data being restricted to a total of 141 hours over the total of 13 days. (No solar wind data were available for Sep 9 2017). The IMF (B_*tot*_) data have fewer gaps, covering a total of 258 hours over 13 days.

**Figure 11:**
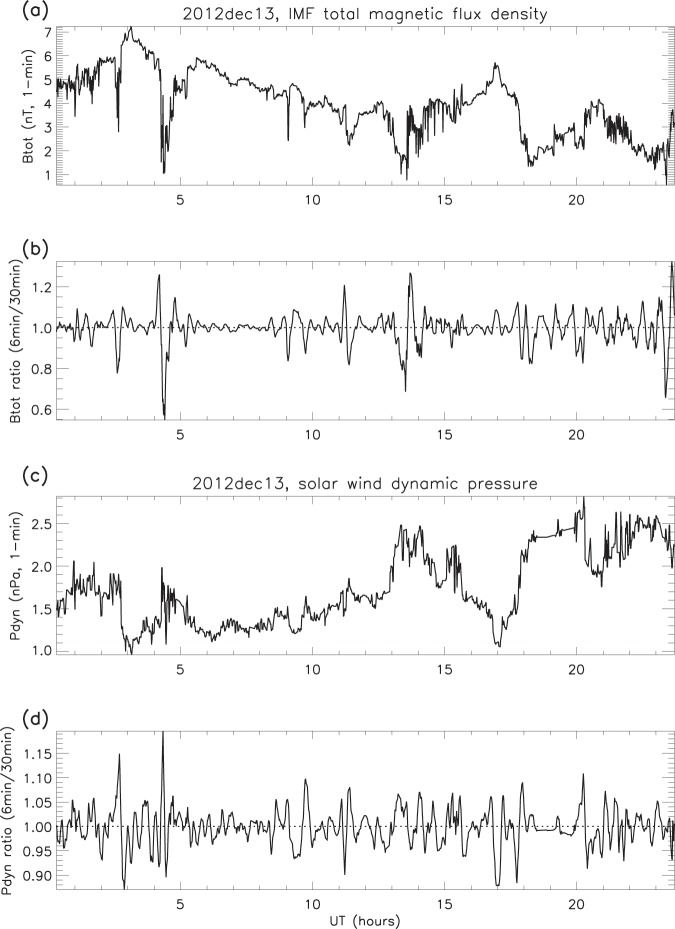
Solar wind and IMF data on December 13 2012: (**a**) IMF total magnetic flux density (B_*tot*_, original time series); (**b**) B_*tot*_ ratios; (**c**) solar wind dynamic pressure (P_*dyn*_, original time series); (**d**) P_*dyn*_ ratios (examples).

**Figure 12:**
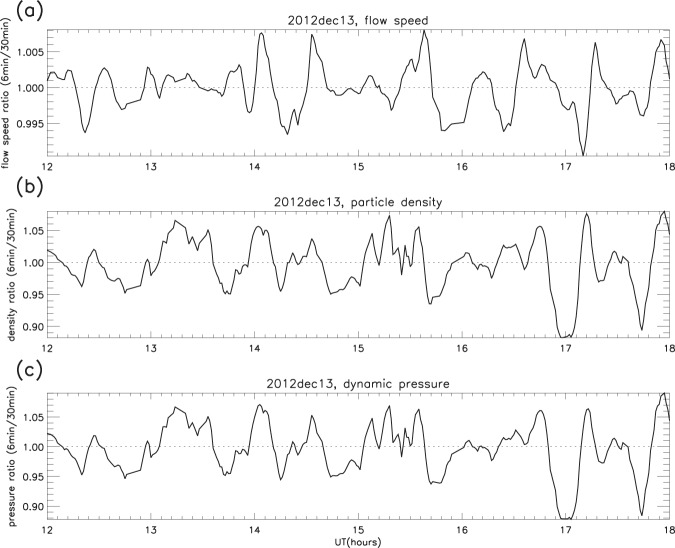
Solar wind particle ripples: (**a**) flow speed, (**b**) particle density, (**c**) dynamic pressure. Dec 13 2012, 1200–1800 UT (example).

**Figure 13:**
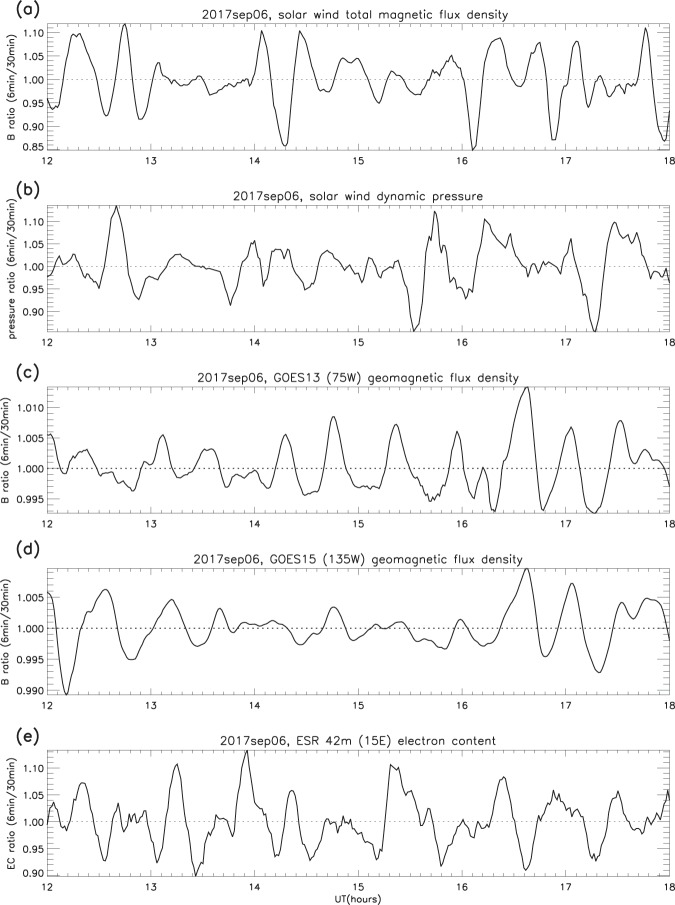
Tracing the ripples from L1 to the F-region: (**a**) SW total magnetic flux density, (**b**) SW dynamic pressure, (**c,d**) GOES 13 and 15 geomagnetic flux density, (**e**) F-region electron content. Sep 6 2017, 1200–1800 UT (example).

The measurements were reduced using the same smoothing procedure (see *Validation of the smoothing procedure*), and examples of the original time series for both total magnetic flux density and solar wind dynamic pressure are compared with their respective ratios in Fig. 11. It is apparent from Figs. 12 and 13 that ripples similar to those in the magnetosphere (GOES) and the F-region (ESR radars) are present in the solar wind. The example in Fig. 12 shows that the ripples in dynamic pressure (proportional to the product of number density and the square of the velocity) are driven more by density than by velocity: for pressure and density the amplitudes and variations are remarkably similar, whereas for pressure and velocity they are not. The second example (Fig. 13) traces the path from L1 to the F-region, so that the ripples in IMF total flux density, solar wind dynamic pressure, geomagnetic field and electron content can be compared. It is clear that similar structures are found in all the parameters, and the same is true throughout all the other days. However, the peaks in the solar wind and geomagnetic field ratios do drift in and out of phase, as was the case with the F-region electron content.

**Figure 14:**
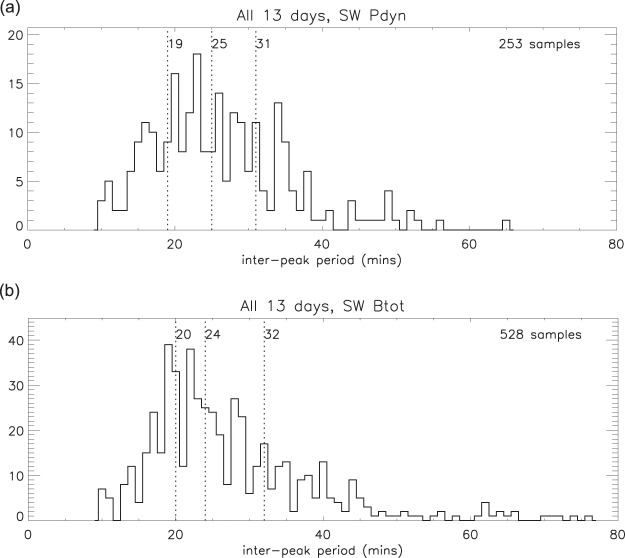
Statistics of the inter-peak periods over all 13 days: (**a**) solar wind dynamic pressure; (**b**) IMF total magnetic flux density.

The ripples in dynamic pressure and IMF flux density are of a similar magnitude (relative to the background) to those in electron content, and clearly-defined peaks with a magnitude of ≥1.01 were selected for each of the 13 days (see *Selection criteria*). Figure 14 shows the statistics of the inter-peak periods for the dynamic pressure and total IMF flux density for all 13 days combined, and in Table 1d the medians and quartiles are summarised for all 13 days and for the separate “seasons”. The similarity between the frequency of the ripples in P_*dyn*_ and B_*tot*_ is clearly evident.

### Summary

Comparisons have been made between the frequency of the ripples in the solar wind (at L1), the geomagnetic field (at 6.6 Re), and the F-region electron content (above Svalbard, and within 670 km towards the north-west). The results, shown in Table 1, provide strong evidence that ripples of period about 25 minutes (frequency 0.7 mHz) which are prevalent in the solar wind (in both the particles and the field) are likely to be the cause of structures of very similar frequency within the magnetosphere and thence the F-region of the ionosphere at high latitude.

In the 14 days of observations, geomagnetic activity reached maximum intensity (K_*p*_ = 8+, A_*p*_ = 236) during the interval 1200–1500 UT on September 8. The ripples in P_*dyn*_ (but not B_*tot*_) increased in amplitude, and B_*z*_ showed a prolonged negative enhancement, resulting in frequent substorm activity. The amplitude of the ripples in the geomagnetic field and the electron content increased accordingly, but there was no significant change in the inter-peak period of any of the parameters (P_*dyn*_, B_*tot*_, GOES13 and 15, 32 m EC, 42 m EC). However, the electron content ripples (both 32 m and 42 m) showed significant disruption, probably resulting from substorm activity.

## Comparisons of Peak Magnitudes

### The ESR 32 m and 42 m peak magnitudes in electron content

**Figure 15:**
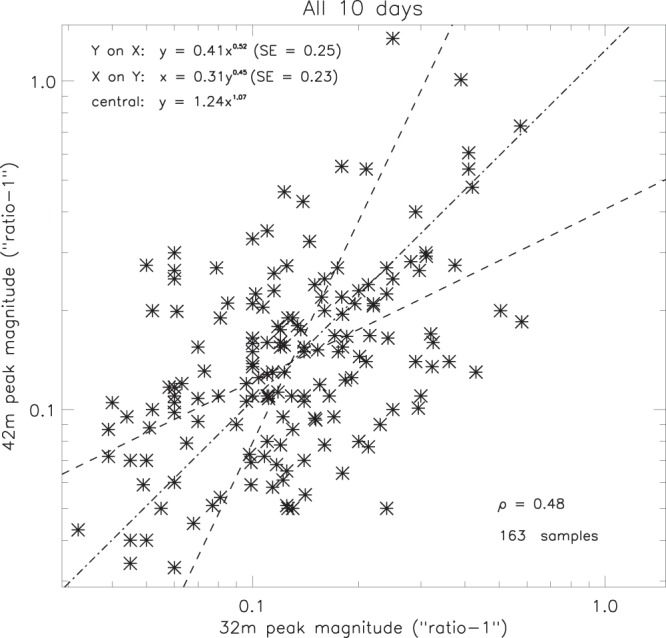
Comparison of magnitudes for selected pairs of associated ESR 32 m and 42 m peaks (SE = standard error).

Though there is close agreement between the 32 m and 42 m electron content periodicities (*Extension using ESR common programme data*), the same is not necessarily true for the peak magnitudes, a comparison of which is shown in Fig. 15. A logarithmic scale was used because the magnitudes are biased towards the lower values, about 30% being less than 1.1. Furthermore, subtraction of unity (“ratio - 1” in the axis labels) gives the peak of the fluctuations as a proportion of the background level. (Subtraction of unity simply reduces the magnitude of the ripples without affecting their structure, as verified in *Validation of the smoothing procedure*.) Though a trend is clearly present, the correlation coefficient (0.48) implies that the peak magnitudes are only weakly related, and that some other process is modulating the amplitude of the ripples without significantly affecting their frequency. However, application of the Fisher z-transform (Table 2) indicates that the probability of this correlation occurring by chance is less than 0.1%, so there is certainly evidence of a relation between the peak magnitudes, the central line of which is given by -

2 $${R}_{42}^{\ast }=1.24{({R}_{32}^{\ast })}^{1.07}$$

where ${R}_{42}^{\ast }$ is the magnitude of the 42 m electron content peaks, and ${R}_{32}^{\ast }$ is the magnitude of the 32 m peaks. (The asterisk notation indicates that unity has been subtracted from the peak magnitude of any given parameter).

#### GOES13 and GOES15

**Figure 16:**
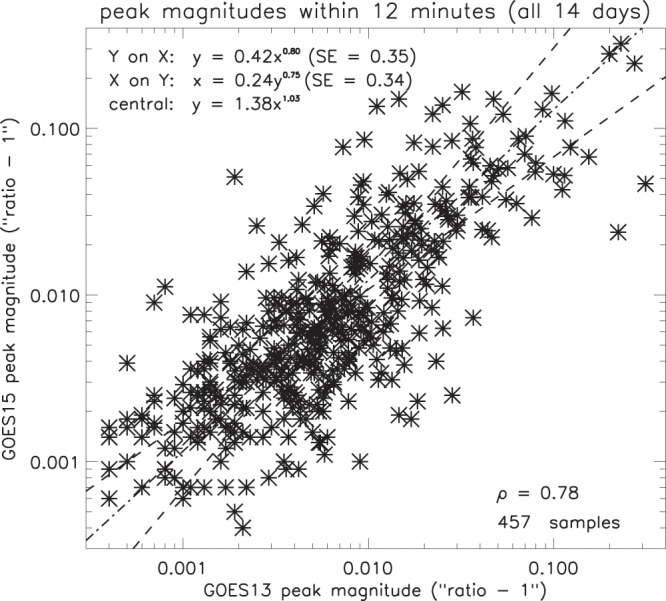
Comparison of magnitudes for selected pairs of associated GOES13 and 15 peaks (SE = standard error).

The GOES13 and 15 peak timings were compared in *Geomagnetic Field Observations*. It is also instructive to compare the magnitudes of closely-associated pairs of peaks, and this relationship is shown in Fig. 16 for peaks separated by less than 12 minutes. As for the peak magnitudes in electron content (above), a logarithmic scale was used and unity was subtracted from the peak magnitudes, most of which are small in relation to the background (about 98% being less than 1.1). There is good correlation (0.78), and a large number of samples (457), indicating that the amplitude of the ripples in the geomagnetic field tends to be uniform over 4 hours of local time. If ${{\rm{G}}}_{13}^{\ast }$ is the magnitude of the peaks in GOES13 flux density and ${{\rm{G}}}_{15}^{\ast }$ is the magnitude of the peaks in GOES15, the central line of the regression (Table 2) is given by -

3 $${G}_{15}^{\ast }=1.38{({G}_{13}^{\ast })}^{1.03}$$

The gradient of the central regression line is very close to unity (1.03), the slight discrepancy possibly implying some degree of bias between the two spacecraft (though whether or not this is instrumental bias is unclear).

### Peak magnitudes within the magnetosphere

#### GOES(13,15) and the geomagnetic activity index A_*p*_

**Figure 17:**
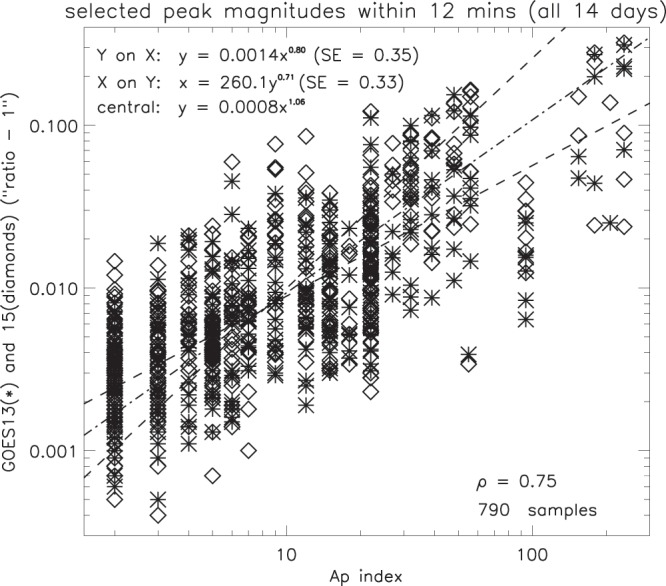
Comparison of magnitudes for selected GOES peaks over a range of geomagnetic activity, as indicated by the A_*p*_ index (SE = standard error). The samples with A_*p*_ > 90 apply only to September 7 and 8.

Geomagnetic activity on the days in question ranged from “quiet” (on Dec 11–14, Feb 27, Mar 24 and Sep 6), to “moderate” (on Feb 28, Mar 1–2, Mar 23), to “severe” (on Sep 7–8). Only Sep 7 and 8 were affected by CME activity, and substorms were only prevalent during these days. The GOES peak magnitudes and their corresponding A_*p*_ indices are compared in Fig. 17, and there is strong evidence of a statistical relation between the GOES peaks and geomagnetic activity (as might be expected), the correlation coefficient being 0.77 for 914 samples. For a given value of Ap, the peaks in the GOES flux density ratio vary by about a factor of 10. If G* is the peak GOES flux density and A_*p*_ is the index of geomagnetic activity, the central line of the regression (Table 2) is given by -

4 $${G}^{\ast }=0.0008{({A}_{p})}^{1.06}.$$

The exponent being very close to unity (1.06), this approximates to a simple linear relation between G* and A_*p*_.

#### GOES and the 42 m electron content

Though there is clearly a connection between GOES and electron content peaks in terms of periodicity, there is no evidence of the same being true for peak magnitudes, the correlation between the GOES and 42 m field-aligned electron content peaks being only 0.07.

### Peak magnitudes in the solar wind: dynamic pressure and IMF total flux density

The flow speed of the solar wind varied in the range 280–850 km/s during the selected days, giving a travel time from L1 to the Earth’s bowshock in the range 30 to 90 minutes. Assuming a 10% error, this means that the timing of the L1-to-bowshock correction is subject to as much as 9 minutes uncertainty. For the purposes of this study, such an uncertainty precludes any useful comparison of the timing of solar wind measurements with either GOES or the ESR radars. However, there are some clearly defined peaks that are separated by no more than about 10 minutes, and their magnitudes have been compared.

#### Solar wind dynamic pressure and geomagnetic flux density

**Figure 18:**
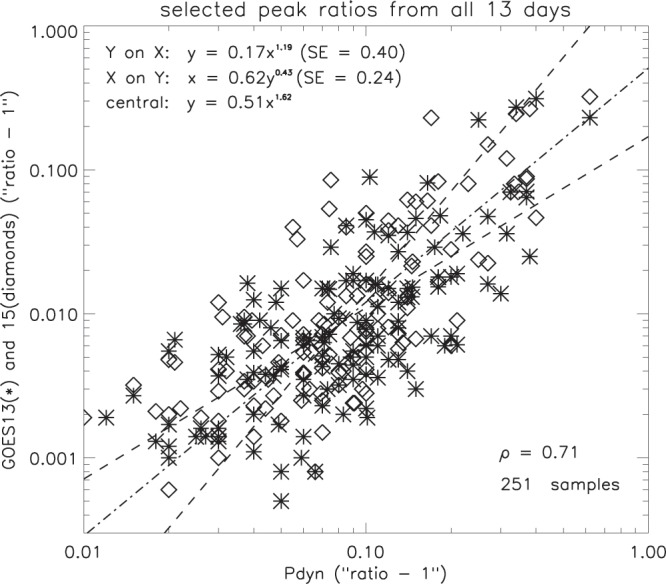
Comparison of magnitudes of selected pairs of GOES and P_*dyn*_ peaks (SE = standard error).

The relation between the magnitudes of clearly associated P_*dyn*_ and GOES peaks is summarised in Fig. 18 for all 13 days. For P_*dyn*_, 57% of the peak magnitudes are less than 1.1, so, as before, a logarithmic scale was used with unity subtracted from the values. Out of the 251 samples, 243 have a time difference of no more than 10 minutes. Only 8 differ by 10–15 minutes, and these were very clearly defined with no other peaks within 30 minutes. The correlation coefficient (0.71) shows a strong association between the amplitude of the ripples in the dynamic pressure of the solar wind and the corresponding response in the geomagnetic field. If G* is the peak GOES flux density and P* is the peak dynamic pressure of the solar wind, the central line of the regression for all times of day (Table 2) is given by -

5 $${G}^{\ast }=0.51{({P}^{\ast })}^{1.62}$$

Tests also indicate that there is no local time dependence between the GOES and P_*dyn*_ peak magnitudes, there being no significant difference between the peak magnitude correlations on the dayside and the nightside.

#### IMF flux density and geomagnetic flux density

**Figure 19:**
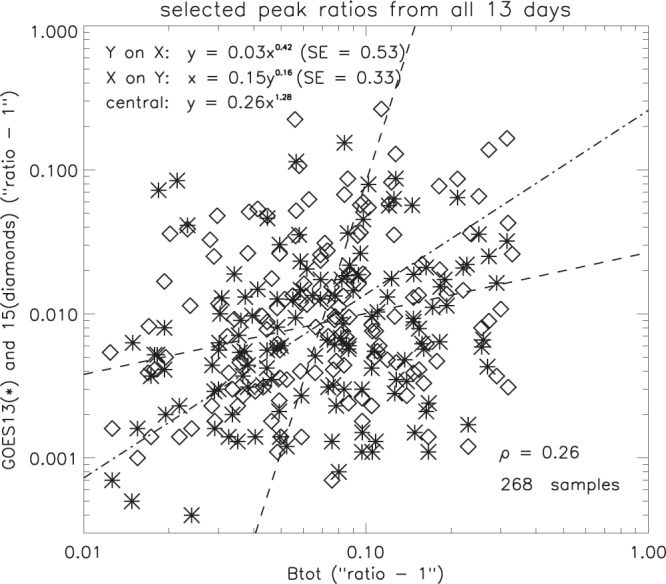
Comparison of magnitudes of selected pairs of GOES and B_*tot*_ peaks (SE = standard error).

The relation between the magnitudes of clearly associated B_*tot*_ and GOES peaks is summarised in Fig. 19, for all 13 days. For B_*tot*_, about 67% of the peak magnitudes are less than 1.1, so a logarithmic scale was used with unity subtracted from the values, as before. Out of the 268 samples, only 3 have a time difference in the range 10–15 minutes, and these were very clearly defined with no other peaks within 30 minutes. The correlation coefficient (0.26) shows a very weak association between the amplitude of the ripples in the IMF total flux density and the corresponding response in the geomagnetic field.

#### P_*dyn*_ and the 42 m electron content

As in the case of GOES, there appears to be no association between the amplitude of the ripples in P_*dyn*_ and those in electron content, the correlation coefficient being only 0.03.

#### Period of maximum activity

In the 14 days of observations, geomagnetic activity reached maximum intensity (K_*p*_ = 8+, A_*p*_ = 236) during the interval 1200–1500 UT on Sep 8. Regarding the magnitude comparisons GOES13-GOES15, GOES1315-Ap, and GOES-P_*dyn*_ (Figs. 16–18 respectively), the values are all grouped together at high values (as expected) during this interval. However, the electron content values in the 32 m–42 m comparison (Fig. 15) are more scattered, probably due to F-region disruption by substorms, though there are only 5 points of comparable pairs (with 2 being at low values).

## Discussion: A Possible Source of the Ripples

There have been reports of ground-based and space-based observations of discrete magnetospheric pulsations since the late 1960s. Herron^2^ produced a power spectrum of persistent geomagnetic variations which showed a peak centred near 0.5 mHz. More recently, Chen and Kivelson^3^, using ISEE-2 magnetometer data from the magnetotail lobes, found a peak in power near 0.48 mHz. Rinnert^4^ observed periodic enhancements with frequencies between 0.3 and 0.4 mHz in EISCAT radar measurements of E-region electron density. Using Prognoz-8 magnetometer data from the magnetotail lobes, Nikutowski *et al*.^5^ found oscillations with frequencies 0.25, 0.5, 0.8, and 1 mHz.

Guo *et al*.^6^ have shown that Alfvén waves originating in the solar wind can cause significant gravity wave disturbances in the Earth’s thermosphere under quiet geomagnetic conditions. However, as part of this study, tests carried out on Alfvén wave and 42 m electron content peak magnitudes, under both quiet (December 12 and 13, 2012) and storm (September 8, 2017) conditions, give a correlation coefficient of only 0.33. It is suggested that the contribution of Alfvén waves to the fluctuations in F-region electron content is limited because the particle density variations in the solar wind are very small. Furthermore, Guo *et al*.^7^ have linked the prolonged multiple excitation of large-scale gravity waves with intermittent southward magnetic fields of Alfvénic fluctuations. Significant auroral electrojet (AE) intensifications were found to have occurred, implying that Alfvénic fluctuations might play a key role in the solar wind-magnetosphere interaction, thus affecting the AE response. In this study, the correlation between the AE index and the electron content peak magnitudes was found to be very weak (*ρ* = 0.0 for the 32 m antenna, 0.19 for the 42 m, 171 samples), though there is a significant correlation (*ρ* = 0.67) between GOES peak magnitudes and AE.

Though it is well known that changes in solar wind dynamic pressure directly affect the magnetospheric field, some studies have explored the possibility that such discrete magnetospheric pulsations might arise directly from oscillatory sources inherent in the solar wind (for example, Kepko *et al*.^8^, Kepko and Spence^9^, Viall *et al*.^10^). Kepko *et al*.^8^ provide evidence that solar wind number density and dynamic pressure fluctuations contain power at discrete frequencies, and that these dynamic pressure oscillations act as direct sources for long-period magnetospheric pulsations, detected at geosynchronous orbit by the GOES spacecraft. Several studies (e.g. Freeman *et al*.^11^, Motoba *et al*.^12^, Sarafopolous^13^) have reported a direct ground response to solar wind dynamic pressure oscillations with periods of a few minutes to tens of minutes. Lessard *et al*.^14^ showed five examples of global magnetospheric oscillations at discrete frequencies below 1 mHz, and suggested that solar wind oscillations were driving the magnetospheric oscillations.

Motoba *et al*.^15^ used a magneto-hydrodynamic simulation to examine the response of the global magnetosphere-ionosphere system to a long-period (10-minute) solar wind dynamic pressure oscillation. The ground geomagnetic field indicated a quasi-sinusoidal waveform with the same period as that of the applied oscillation in dynamic pressure.

Viall *et al*.^10^ conducted a long-term statistical analysis of the distributions of significant frequencies of periodic solar wind number density structures and dayside magnetospheric oscillations in the 0.5–5.0 mHz range. The enhancements in the distribution of magnetospheric frequencies (at 0.2, 0.8, 1.7 and 2.8 mHz) are similar to those in the solar wind, implying that the magnetosphere is often directly driven.

Matteo *et al*.^16^ reported solar wind fluctuations with frequencies 0.7, ≈1.4, ≈2.0, and ≈4.8 mHz, similar to those reported by Viall *et al*.^10^. However, they suggest that a more extended statistical analysis is needed using alternative analytical methods in order to provide more evidence for the existence of specific solar wind fluctuation frequencies.

The fluctuations investigated here are components of variation in the geophysical quantities being observed. They are also quite small, in some cases rarely exceeding 1% of the background quantity, yet their persistence, and the level of agreement between the various quantities (electron content at high latitude, geomagnetic field intensity at geosynchronous orbit, and the dynamic pressure of the solar wind at L1) implies that that they are significant features of Earth’s geophysical environment.

Kepko *et al*.^8^ reported oscillations of frequency 0.7 mHz (period 24 mins) in observations of both solar wind dynamic pressure and geosynchronous magnetic field strength, and suggested that one possible source of these oscillations is instabilities generated in the solar wind during its passage through interplanetary space. However, the ripples that we have detected occur in different years and seasons, and are so regular in frequency that they appear to be a permanent property of the solar wind, so it seems unlikely that instabilities are the cause because they would be associated more with stochastic phenomena.

A second possibility is that the ripples are the result of solar oscillations. The power spectrum of charged particle fluxes detected by the Ulysses spacecraft often contains periodic components that are not simply harmonics of solar rotation (Lanzerotti *et al*.^17^). Thomson *et al*.^18^ noted that the observed frequencies were consistent with the frequency range of solar pressure (p-)modes, and solar gravity (g-)modes. Bertello *et al*.^19^ determined 11 oscillatory modes, of which three corresponded to frequencies 0.5, 0.8, and 1.0 mHz (periodicities 31, 20 and 17 minutes). Similar frequencies were subsequently reported at 1 AU in ACE data and beyond 1 AU in Ulysses data (Thomson *et al*.^20^). Furthermore, Thomson *et al*.^21^ showed that the modes are coherent and are preserved between ACE and Ulysses. Low-frequency *p* and *g* modes occur in the range 0.1 to 2.0 mHz (Provost *et al*.^22^), and our results (median 0.7 mHz) are clearly within this range.

Though relating terrestrial geomagnetic pulsations to solar oscillations is an intriguing possibility, subsequent work addressing whether the solar wind contains imprints of solar oscillation modes has yielded conflicting results (Kepko *et al*.^8^).

Recent studies have reported the presence of quasi-periodic density structures in the solar corona (Viall and Vourlidas)^23^, in the solar wind from 0.3 to 0.6 AU (Di Matteo *et al*.)^24^, and at L1 (Kepko et al.)^25^. These periodicities are in the range 60–130 mins, and our results (25–27 minutes) do not compare favourably. However, Kepko and Viall^26^ present several examples of magnetospheric ultra-low frequency pulsations associated with stream interaction regions and demonstrate that the observed magnetospheric pulsations were also present in the solar wind number density. The density structures lead to a quasi-static and globally coherent “forced-breathing” of Earth’s dayside magnetospheric cavity affecting both the geomagnetic field and energetic particles (even at high latitudes), with periods of about 20 minutes, similar to those described in this study.

## Validation of Methods

### Validation of the smoothing procedure

Tests were conducted to ensure that the emergence of the ripples was not an artifact of the smoothing method by: (1) varying the width of the smoothing windows, both numerator and denominator, (2) testing the utility of spectral analysis as an alternative method for extraction of the ripples in the frequency domain, and (3) quantifying the effect of applying the method to random data, such as that derived from radioactive decay.

#### Variation of the smoothing windows

**Figure 20:**
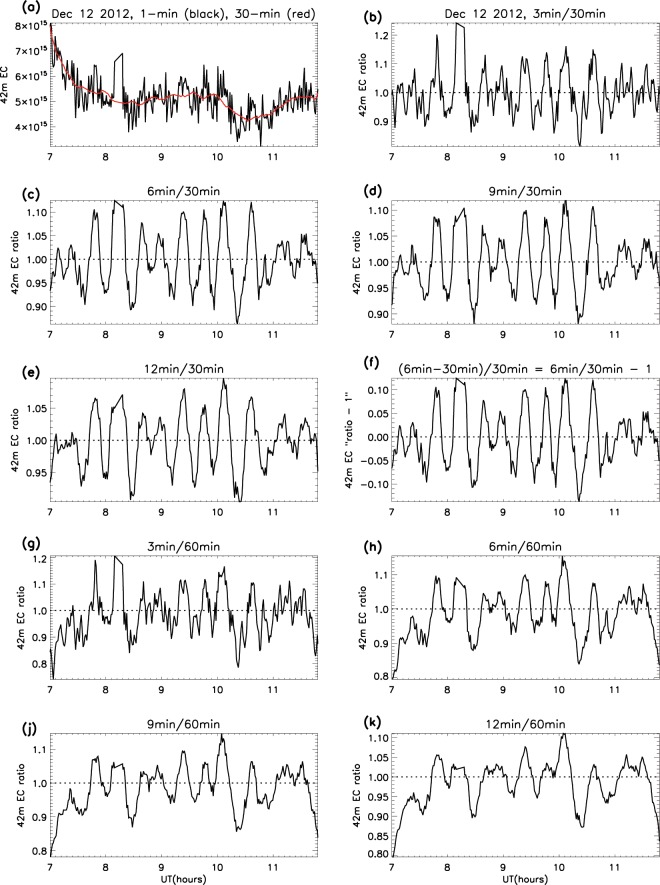
Example of the smoothing method, showing: (**a**) the raw 1-minute EC data (with 30-minutes moothing in red); (**b–e**) EC ratios with 3, 6, 9 and 12 minute smoothing intervals and 30 minute divisor; (**f**) verification of unity subtraction in *Comparisons of Peak Magnitudes*; (**g–k**) the same smoothing intervals with 60 minute divisor.

The example in Fig. 20 shows the 42 m electron content ratios on a selected day, but the same principle applies to all the various parameters on all days. The features in the raw data emerge when the background (smoothed to 30 and 60 minutes) is divided into the same data smoothed at intervals of 3, 6, 9 and 12 minutes. This supports the conclusion that the variations being studied are real, and were not generated by the smoothing procedure (the features are less apparent using the 60 minute divisor, but are visible nonetheless). In addition, the technique of subtracting unity in *Comparisons of Peak Magnitudes* is verified by comparing Fig. 20c,f: the profiles are identical, but all values are reduced by unity.

A similar smoothing procedure was used by Guo *et al*.^6^, in which a “bandpass filter” is described. This involved 4 and 25 minute averaging to extract fluctuations in magnetospheric gravity waves, but the general approach is the same as the smoothing method used in this study.

#### Frequency spectrum using fourier analysis

The Fourier spectra of the 1 minute observations for each of the 14 days show no clear peaks at any frequencies. It is suggested that this is because the signal to noise ratio is too low for a simple Fourier analysis to be able to identify them, though more sophisticated techniques would probably be more effective. However, our “bandpass filter” method also reveals the variation in peak magnitudes, which would not be the case using a spectral approach.

#### Smoothing method applied to random number data

**Figure 21:**
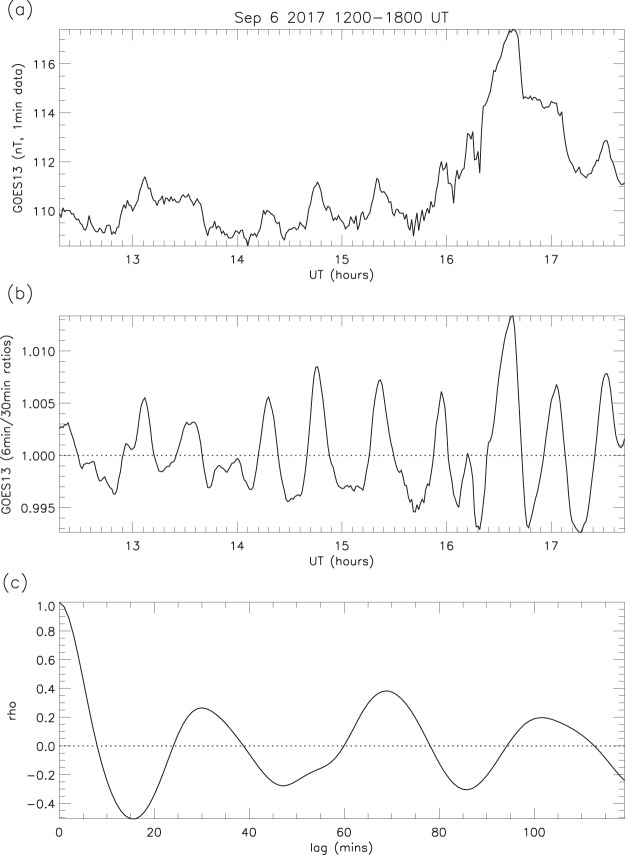
GOES13 observations (Sep 6 1200–1800 UT): (**a**) 360 1-min values; (b) 6 min/30 min ratios; (**c**) auto-correlation of ratios (0–120 min lags).

**Figure 22:**
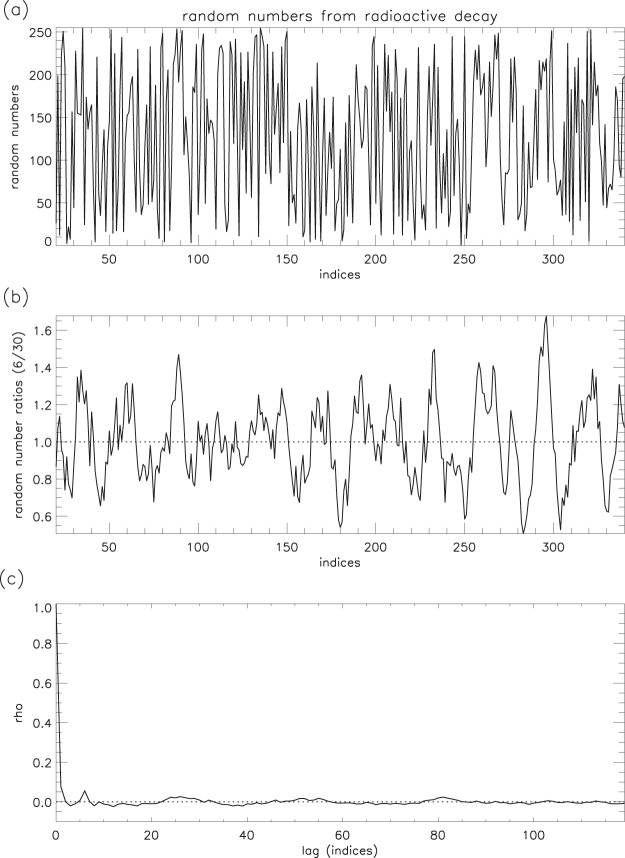
Random numbers from radioactive decay: (**a**) 360 samples; (**b**) 6/30 ratios of samples; (**c**) auto-correlation of ratios (0–120 lags).

Auto-correlation tests were carried out on both the observed ratios and those derived from random numbers generated from radioactive decay. The profiles of the correlograms are quite different in the two cases (Figs. 21 and 22), further supporting the conclusion that the ripples in the observations are real, and are not generated by the smoothing method.

### Selection criteria

The 6 min/30 min ratio time-series of all parameters were generally well-defined, smooth structures, and peaks could therefore be selected without ambiguity. However, where necessary, the following selection criteria were imposed.

#### Magnitude limits

In the case of the ESR EC data (32 m and 42 m), the peaks were not well-defined below a ratio limit of 1.03, and were therefore excluded. The same applied to the solar wind data below 1.01. However, no such limit was necessary in the case of the geomagnetic data, the peaks being well structured to the lowest levels. These peak magnitude limits (Table 3) were chosen by inspection of the overall data set.

Conversely, a peak was considered to be a separate structure from that preceding it if the intervening dip dropped below the lower limit (see *Structures comprising multiple peaks*).

#### Associations between peaks in different parameters

Associations between peaks in different parameters were restricted to time differences of no more than ±12 minutes to avoid any uncertainty in the comparisons. In some cases the time difference increased to ±15 minutes, but in these exceptions there was no ambiguity.

#### Structures comprising multiple peaks

In a few instances, a structure comprised multiple peaks. In such cases, if a dominant peak was present, then that peak was assumed to define the group. However, if no dominant peak was present, the centre of the group was selected as the peak UT. A peak was considered to be separate from a group structure if the intervening dip dropped below the lower selection limit (see *Magnitude limits*). In exceptional cases, a side spur was assumed to be part of a dominant structure.

## Summary

- Previous studies have estimated the frequency of the ripples in the solar wind and geomagnetic field parameters by analysing the power spectrum of the raw data. In this paper we have used a bandpass filter to reveal the ripples themselves, thus making it possible not only to determine their frequency, but also to correlate their peak magnitudes and to compare, where applicable, the time differences between the various parameters. By this method we have provided further evidence for a connection between the ripples in the solar wind, the geomagnetic field and the ionosphere.
- Ripples of median periodicity 25–27 minutes observed in the high-latitude F-region electron content (in the vicinity of Svalbard) and in the geomagnetic field (at geosynchronous orbit at 6.6 Re) show a strong relationship with ripples of similar periodicity in the solar wind particle pressure and IMF at L1.
- The ripples were observed in different years and seasons, so they appear to be a persistent feature of the solar wind, the magnetosphere, and the F-region at high latitude.
- The ripples in the F-region electron content occur primarily within the altitude range 230–365 km above Svalbard (42 m antenna) and extend about 670 km towards 29°W of true N (32 m antenna).
- The peaks in the 32 m and 42 m electron content occur within 5 minutes of each other in 50% of cases, and there seems to be no local time dependency. The peak magnitudes, however, are only weakly correlated (*ρ* = 0.48, 163 samples).
- The ripples in the geomagnetic field precede those in the electron content by about 2 minutes in the median.
- The ripples in the geomagnetic field are spatially and temporally persistent, and exhibit greater timing coherence on the dayside than on the nightside, supporting the conclusion that the solar wind is the driving mechanism (and therefore, by implication, that it does not involve the substorm process).
- The inter-quartile range of the ripples shows a gradual increase from the solar wind (11–12 minutes), to the geomagnetic field (14–15 minutes), to the F-region (17–18 minutes), indicating a broadening of the variation in periodicity as the effect propagates within geospace.
- The peak magnitudes of the ripples in GOES13 and GOES15 are strongly correlated (*ρ* = 0.78, 457 samples), indicating that the amplitude of the ripples in the geomagnetic field is consistently uniform over 4 hours of local time.
- The peak magnitudes of the ripples in GOES are strongly correlated with the A_*p*_ index (*ρ* = 0.77, 790 samples, A_*p*_ > 0). Though this is not unexpected, it is nonetheless a confirmation that the amplitude of the ripples in the geomagnetic field (not the background) is a good indication of the level of geomagnetic activity.
- The ripples in the solar wind are exhibited by both the particles (represented by the dynamic pressure, in particular the number density) and the total (or scalar) IMF, and they drift in and out of phase over periods of a few hours.
- The peak magnitudes of the ripples in GOES and P_*dyn*_ are strongly correlated (*ρ* = 0.71, 251 samples), whereas those between GOES and B_*tot*_ are only very weakly correlated (*ρ* = 0.26, 268 samples). It is therefore evident that the dynamic pressure, not the IMF, is the main driving parameter of the ripples in the geomagnetic field.
- Though it has been demonstrated that there is clearly a connection in terms of periodicity between the ripples in electron content and those in both the geomagnetic field and the solar wind, there is no evidence of the same being true for peak magnitudes (the correlation coefficients being <0.1). This suggests that the amplitude of the ripples in electron content is being affected by some other local factor, as yet unknown.
- It is not unlikely that low-frequency p- and g-mode solar oscillations are the cause of the ripples in the solar wind, and thence the geomagnetic field and the high-latitude F-region.

**Table 3:** Magnitude ranges and selection limits for each parameter.

Parameter	Magnitude range	Selection limits (upper and lower)
32 m EC	1.03–1.60	1.0 ± 0.03
42 m EC	1.03–2.08	1.0 ± 0.03
GOES	1.0004–1.3000	no selection limit imposed
P_*dyn*_	1.01–1.60	1.0 ± 0.01
B_*tot*_	1.01–1.30	1.0 ± 0.01

## Data Availability

All data used in this study are available from the authors, or from sources already specified within the manuscript which, in summary, are: Random numbers: http://www.fourmilab.ch/hotbits/ Solar wind at L1: http://cdaweb.gsfc.nasa.gov/ GOES: https://satdat.ngdc.noaa.gov/sem/goes/data/avg/ ESR: https://www.eiscat.se/schedule/schedule.cgi.
